# From Dysbiosis to Vascular Disease: the Gut Microbiota in Type 2 Diabetes and Atherosclerosis

**DOI:** 10.1007/s11883-026-01485-1

**Published:** 2026-11-09

**Authors:** Sabitha Sasidharan Pillai, Ambika P. Ashraf

**Affiliations:** 1 Center for Endocrinology, Diabetes and Metabolism, Children’s Hospital Los Angeles, Los Angeles, CA USA; 2 Department of Pediatrics, Keck School of Medicine, University of Southern California, Los Angeles, CA USA; 3 Department of Pediatrics, The University of Alabama at Birmingham, Birmingham, AL United States of America

**Keywords:** Gut microbiota, Gut microbiota dysbiosis, Type 2 diabetes, Atherosclerosis, Microbial metabolites

## Abstract

**Purpose of Review:**

Type 2 diabetes (T2D) and atherosclerotic cardiovascular disease (ASCVD) are closely linked chronic disorders and leading causes of morbidity and mortality worldwide. Increasing evidence identifies the gut microbiota (GM), including its microbial genes and metabolites, as potential modifiers of these processes. This narrative review summarizes mechanistic, translational, and clinical evidence linking GM dysbiosis with T2D and atherosclerosis and evaluates microbiota-directed therapeutic strategies.

**Recent Findings:**

Converging mechanistic, translational, and clinical evidence supports GM dysbiosis as a plausible shared contributor to T2D and atherosclerosis through interconnected pathways involving impaired intestinal barrier integrity and metabolic endotoxemia, TLR4/NF-κB- and inflammasome-mediated inflammation, and microbial metabolites, including short-chain fatty acids (SCFAs), bile acids (BAs), trimethylamine N-oxide (TMAO), phenylacetylglutamine (PAGln), and tryptophan-derived indoles. These pathways can influence glucose and lipid metabolism, insulin signaling, immune activation, endothelial function, and vascular inflammation, providing a biological framework linking dysglycemia with increased cardiovascular risk and supporting the GM as a potential therapeutic target. However, the field remains limited by predominantly observational and confounded human data, incomplete causal evidence, and heterogeneous responses to microbiota-directed interventions. Dietary modification, prebiotics, probiotics, synbiotics, next-generation probiotics and postbiotics, fecal microbiota transplantation, and microbiota-modulating antidiabetic therapies have shown signals of benefit on metabolic or vascular surrogate endpoints, but evidence for durable clinical benefit remains limited and inconsistent across strains, populations, and study designs.

**Summary:**

Future progress in GM manipulation to improve cardiometabolic outcomes will require function-focused, causally grounded research and adequately powered, outcome-driven clinical trials incorporating GM-guided patient stratification. Until such evidence is available, the GM should be viewed as a biologically compelling and promising, but not yet clinically actionable, link between metabolic and atherosclerotic disease.

## Introduction

Type 2 diabetes (T2D) and atherosclerotic cardiovascular disease (ASCVD) are closely linked chronic disorders and leading causes of morbidity and mortality worldwide [1–3]. T2D accelerates atherosclerosis through insulin resistance (IR), hyperglycemia, atherogenic dyslipidemia, oxidative stress, endothelial dysfunction, inflammation, and thrombosis. Increasing evidence identifies gut microbiota (GM), the intestinal community of bacteria, archaea, fungi, and viruses and their collective genes and metabolites, as a potential modifier of these processes [4, 5]. This narrative review integrates the current mechanistic, translational, and clinical evidence linking gut microbiota dysbiosis with both T2D and atherosclerosis, with particular emphasis on shared pathways and microbial metabolites that may contribute to metabolic and vascular disease. We also critically evaluate the evidence for microbiota-directed therapeutic strategies and highlight the limitations that currently prevent translation into routine clinical practice. By bringing these areas together, the review identifies important gaps in the field and outlines priorities for future research, particularly the need to move from associations and surrogate biomarkers toward causal evidence and clinically meaningful cardiovascular and metabolic outcomes.

### Methodology

A nonsystematic literature search was conducted using PubMed (from database inception) and Google Scholar through August 2026. The search included the following combinations of keywords: “gut microbiota” AND “type 2 diabetes”; “gut microbiota” AND “atherosclerosis”; and “gut microbiota” AND “type 2 diabetes” AND “atherosclerosis.” Additional relevant studies were identified from the reference lists of selected articles. We emphasized human studies, meta-analyses, and interventional trials, while preclinical studies were included primarily to provide mechanistic context. Given the heterogeneity of study designs, populations, interventions, and outcomes, studies were selected based on relevance and methodological rigor rather than according to formal systematic-review criteria. The characteristics of the randomized clinical trials and of the observational studies discussed in this review are summarized in Tables 1 and 2, respectively [6–34].

**Table 1:** Characteristics of randomized clinical trials of microbiota- and metabolite-directed interventions. Interventions span dietary, microbial, metabolite, pharmacologic and surgical approaches. Findings concern metabolic or vascular surrogates unless otherwise specified

Study and country	Design and duration	Population	Intervention vs comparator	Microbiota or metabolite assessment	Metabolic outcomes	Lipid or vascular outcomes	Key limitation
Vrieze, et al 2012 [13] * Netherlands	Randomized, controlled mechanistic pilot; 6 weeks	18 men with metabolic syndrome	Single lean-donor intestinal FMT vs autologous FMT	Yes: fecal microbiota profiling	Peripheral insulin sensitivity improved at 6 weeks; associated enrichment of butyrate producers	No vascular event endpoint	Very small male-only study; short follow-up; published correction in 2013
Firouzi, et al 2017 [7] Malaysia	Double-blind, placebo-controlled; 12 weeks	136 randomized; T2D; age 30–70 years	Six-strain Lactobacillus and Bifidobacterium mix vs placebo	Yes: targeted fecal Lactobacillus and Bifidobacterium quantification	HbA1c benefit in PP, not ITT; fasting insulin improved in both analyses	No significant lipid, blood-pressure or hsCRP effect	Attrition and PP-dependent HbA1c result; targeted counts do not characterize the whole microbiome
Mobini, et al 2017 [12] Sweden	Double-blind, placebo-controlled; 12 weeks	46 adults with insulin-treated T2D; age 50–75 years	L. reuteri DSM 17938, 10^8^ or 10^10^ CFU/day, vs placebo	Yes: fecal microbiota and serum bile acids	No HbA1c benefit; exploratory insulin-sensitivity signal in a subset	No demonstrated vascular or cardiovascular event benefit	Small three-arm study; subgroup response should not supersede the null primary finding
Kootte, et al 2017 [15] * Netherlands	Randomized, controlled; assessment at 6 and 18 weeks	38 men with metabolic syndrome	Lean-donor FMT (n = 26) vs autologous FMT (n = 12)	Yes: fecal microbiota and plasma metabolomics	Insulin sensitivity improved at 6 weeks, not 18 weeks; baseline microbiota associated with response	No vascular event endpoint	Small male-only cohort; transient effect; exploratory responder signatures
Wu, et al 2017 [21] Sweden	Double-blind, placebo-controlled; 4 months	40 adults with treatment-naive T2D	Metformin 1,700 mg/day (n = 22) vs placebo (n = 18)	Yes: shotgun metagenomics and functional analyses	Metformin improved glycemia and altered microbial composition/functions	No vascular event endpoint	Small trial; mouse-transfer experiments support plausibility but do not quantify microbial mediation in humans
Gu, et al 2017 [22] China	Randomized, active-controlled; 3 months	106 randomized; 94 analyzed; treatment-naive T2D	Acarbose vs glipizide	Yes: shotgun metagenomics and plasma bile acids	Similar glucose lowering, but acarbose produced larger microbiome and bile-acid changes	Acarbose-associated lipid responses varied with baseline microbiota; no vascular event endpoint	Completer analysis; active-drug comparison cannot isolate microbial mediation
Smits, et al 2018 [14] * Netherlands	Double-blind, randomized pilot; 2 weeks	20 men with metabolic syndrome	Single lean vegan-donor FMT vs autologous FMT	Yes: fecal microbiota and carnitine/choline-derived TMAO production	No established metabolic treatment benefit	No improvement in TMAO production capacity or aortic ^18^F-FDG uptake	Small, short male-only study; vascular imaging is a surrogate
Zhao, et al 2018 [6] China	Randomized, open label; 12 weeks	43 adults with T2D	High-fiber diet (whole grains, medicinal foods and prebiotics) vs usual dietary advice; both groups received acarbose	Yes: shotgun metagenomics and SCFAs	Greater HbA1c improvement with enrichment of selected SCFA-producing strains	No vascular or cardiovascular event endpoint	Small trial; complex diet and concurrent acarbose prevent attribution to fiber or microbiota alone
Hsieh, et al 2018 [10] Taiwan	Double-blind, placebo-controlled; 6 months, then 3-month washout	74 randomized; 68 completed; T2D; age 25–70 years	Live L. reuteri ADR-1 (4 × 10^9^ CFU/day) or heat-killed ADR-3 (2 × 10^10^ cells/day) vs placebo	Yes: targeted fecal qPCR	ADR-1 reduced HbA1c; ADR-3 did not show an overall HbA1c benefit; responder analyses exploratory	Total-cholesterol signal with ADR-1; no vascular endpoint	Small three-arm study; completer and post hoc microbial-response analyses
Depommier, et al 2019 [17] † Belgium	Double-blind, placebo-controlled pilot; 3 months	40 randomized; 32 completed; overweight/obese, insulin-resistant adults	Live or pasteurized A. muciniphila (10^10^ bacteria/day) vs placebo	Yes: fecal microbiota profiling	Pasteurized preparation improved insulin sensitivity and reduced insulinemia	Pasteurized preparation reduced total cholesterol; no vascular event endpoint	Exploratory, small completer cohort; not an established T2D/ASCVD population
Horvath, et al 2020 [11] Austria	Double-blind, placebo-controlled pilot; 6-month treatment, 12-month follow-up	26 adults with obesity and T2D; age > 18 years	Nine-strain probiotic (~ 1.5 × 10^10^ CFU/day) plus GOS/FOS-based prebiotic (8 g active/day) vs matched placebos	Yes: fecal microbiome profiling	No improvement in HbA1c or mixed-meal glucose responses	Lp(a) decreased at 6 months; other lipid results largely null	Small pilot, multiple secondary outcomes and background treatment; Lp(a) finding not an event benefit
Ng, et al. 2022 [16] Hong Kong, China	Double-blind, three-arm RCT; 24-week follow-up	61 adults with obesity and T2D	Repeated FMT plus lifestyle intervention, FMT alone, or sham plus lifestyle intervention; every 4 weeks through week 12	Yes: metagenomic assessment of donor engraftment	Sustained engraftment was the primary result; no established durable glycemic efficacy	Favorable lipid/liver-stiffness signals with combined treatment; no ASCVD event endpoint	Small groups; lifestyle co-intervention; engraftment is not a clinical efficacy endpoint
Haghikia, et al 2022 [19] † Germany	Double-blind, placebo-controlled; 8 weeks	62 randomized; 58 analyzed (29 per group); adults with LDL-C > 115 mg/dL	Propionate 500 mg twice/day vs placebo	Yes: fecal microbiota profiled in a subset, with no change in composition or diversity; metabolite-directed intervention	Glycemic efficacy was not the trial focus	LDL-C fell 15.9 vs 1.6 mg/dL (P=.016); total and non-HDL cholesterol also decreased	Lipid surrogate trial in hypercholesterolemia; human plaque regression/event reduction not tested
Coppola, et al 2022 [20] § Italy	Quadruple-blind, placebo-controlled; 6 months	54 children with obesity; age 5–17 years; mean 11 years	Sodium butyrate 20 mg/kg/day (maximum 800 mg/day) vs placebo, both with standard lifestyle care	Yes: shotgun metagenomics	BMI-SDS decrease ≥ 0.25: 96% vs 56% (ITT); insulin/HOMA-IR benefits in PP	No demonstrated lipid or vascular benefit	Pediatric obesity, not T2D/ASCVD; 48 completed; metabolic secondary results were PP
Wortelboer, et al 2023 [18] * Netherlands	Double-blind, placebo-controlled pilot; 28 days	24 adults with metabolic syndrome	Lean-donor sterile fecal filtrate via nasoduodenal tube (n = 12) vs placebo (n = 12)	Yes: bacteriome and phageome profiling	OGTT glucose AUC, HbA1c and HOMA-IR were unchanged; limited exploratory CGM signal	Cholesterol and blood pressure unchanged; no vascular event endpoint	Small, short trial; filtrate contains more than phages; not proof of purified phage efficacy
Zikou, et al 2023 [9] Greece	Double-blind, placebo-controlled; 6 months	91 adults with T2D; mean age 65.1 years	LactoLevure, one capsule/day: L. acidophilus, L. plantarum, B. lactis and S. boulardii, vs placebo	Yes: 16 S rRNA sequencing in a subgroup	Lower HbA1c and fasting glucose vs placebo	Total cholesterol decreased; no established vascular benefit	Microbiome subgroup; no overall diversity difference; treatment changes led to exclusion from further analysis
Peng, et al 2024 [8] ‡ China	Double-blind, controlled; 16 weeks	213 randomized; T2D; age ≥ 18 years; mean ≈62 years	Lactobacillus beverage, 25 mL four times/day (> 10^8^ CFU/mL), vs inactivated Lactobacillus beverage	No: fecal bacterial load and SCFA changes were not measured	No significant between-group HbA1c, fasting-glucose or insulin-resistance benefit in ITT/PP	No significant between-group lipid benefit	Potentially active inactivated-bacteria comparator; baseline metformin imbalance; 172 completed week 16
Olsson, et al 2026 [23] Norway	Microbiome subanalysis of randomized Oseberg trial; 12-month sampling, 5-year remission assessment	77 adults with obesity and T2D in paired-sample subset	Roux-en-Y gastric bypass (n = 39) vs sleeve gastrectomy (n = 38)	Yes: fecal whole-genome sequencing	Microbial richness/functions and surgery-specific changes associated with metabolic improvement and T2D remission	No microbiome-mediated vascular outcome demonstrated	Selected trial subset; microbiota–outcome analyses remain observational despite randomized surgery

*Abbreviations*: ^18^*F-FDG* 18F-fluorodeoxyglucose, *ACS*, Acute coronary syndrome, *ASCVD* Atherosclerotic cardiovascular disease, *AUC* Area under the curve, *BMI* Body mass index, *CCTA* Coronary computed tomography angiography, *CFU* Colony-forming units, *CGM* Continuous glucose monitoring, *CVD* Cardiovascular disease, *FINRISK* Finnish population survey on risk factors for chronic noncommunicable diseases, *FMT* Fecal microbiota transplantation, *FOS* Fructooligosaccharides, *GOS* Galactooligosaccharides, *HbA1c* Glycated hemoglobin, *HOMA-IR* Homeostasis model assessment of insulin resistance, *hsCRP* high-sensitivity C-reactive protein, *IHD* Ischemic heart disease, *ImP* Imidazole propionate, *ITT* intention-to-treat, *LDL-C* low-density lipoprotein cholesterol, *Lp(a)* lipoprotein(a), *MACE* major adverse cardiovascular events, *MetaCardis* Metagenomics in Cardiometabolic Diseases, *MI* Myocardial infarction, *OGTT* oral glucose tolerance test, *PAGln* phenylacetylglutamine, *PP* per-protocol, *qPCR* quantitative polymerase chain reaction, *RCT* Randomized controlled trial, *SCAPIS* Swedish Cardiopulmonary Bioimage Study, *SCFA* Short-chain fatty acid, *SDS* standard deviation score;*T2D* type 2 diabetes, *TMAO* trimethylamine N-oxide

Selection notes: * Landmark mechanistic trial retained despite follow-up shorter than 8 weeks. † Related at-risk population outside the core T2D/metabolic syndrome/ASCVD scope. ‡ Larger informative null trial retained despite absence of microbiota/metabolite measurements. § Pediatric obesity trial included only as contextual metabolite-directed evidence; not evidence for treating adult T2D or ASCVD. Sample sizes denote randomized participants unless otherwise specified. Reported benefits refer to between-group comparisons unless stated otherwise. The randomized bariatric-surgery comparison does not randomize microbial mediation. No listed trial establishes a reduction in ASCVD events attributable to microbiota modification. Sterile filtrate is not equivalent to purified phage therapy. Engineered organisms and microbial enzyme inhibition remain outside the human RCT evidence represented here. The Vrieze, Kootte and Smits trials arose from the same Amsterdam fecal microbiota transplantation program and share investigators and overlapping recruitment; their participants must not be counted as independent evidence

**Table 2:** Characteristics of observational studies linking gut microbiota and microbial metabolites with type 2 diabetes and atherosclerosis

Study and setting	Design and follow-up	Population	Microbial exposure and methods	Main finding	Confounders and analytic context	Key limitation
Qin 2012 [24] China	Two-stage cross-sectional metagenome-wide association study	345 adults with or without T2D	Deep fecal shotgun metagenomics; microbial genes and functional modules	T2D associated with reduced abundance of several butyrate producers and enrichment of opportunistic organisms/functions	Medication, diet and adiposity can influence disease signatures	Cross-sectional association; no temporality or treatment inference
Karlsson 2013 [25] Sweden	Cross-sectional population-based comparison	145 European women with normal, impaired or diabetic glucose control	Fecal shotgun metagenomics and microbial functional profiling	Microbial features discriminated glucose-control categories; signatures differed from those in Chinese cohorts	Age, sex, geography, treatment and metabolic phenotype affect transportability	Female-only cohort; cross-sectional classifiers are not validated incident-disease predictors
Tang 2013 [34] United States	Prospective clinical cohort; 3-year follow-up	4,007 adults undergoing elective coronary angiography	Fasting plasma TMAO by mass spectrometry; adjudicated death, MI or stroke	Higher TMAO associated with incident MACE after multivariable adjustment	Traditional risk factors and renal function considered; separate dietary/antibiotic challenge supported microbial contribution	Referral cohort: diet and kidney clearance can affect TMAO; lowering TMAO was not tested
Forslund 2015 [26] Denmark, Sweden and China	Cross-cohort reanalysis plus additional cohort	784 available human fecal metagenomes spanning diabetes/treatment groups	Shotgun metagenomics with explicit metformin exposure stratification	Metformin strongly confounded T2D-associated signatures; untreated T2D retained depletion of butyrate-producing taxa	Drug exposure was a central analytic variable, rather than an assumed disease effect	Observational drug comparisons; overlap with earlier cohorts limits independence
Jie 2017 [29] China	Cross-sectional case–control metagenome-wide association study	218 adults with ASCVD and 187 healthy controls	Fecal shotgun metagenomics; species/strain and functional profiling	ASCVD associated with enrichment of Enterobacteriaceae and oral-associated species and depletion of selected butyrate producers	Established disease, medications, diet and comorbidities can shape case–control differences	Healthy controls do not separate ASCVD-specific effects from dysmetabolism or treatment
Koh 2018 [28] Netherlands and Sweden	Cross-sectional human discovery/validation with separate experimental studies	Discovery: 5 adults with T2D and 10 BMI-matched controls; validation: 649 adults, age 50–64 years	Portal/peripheral metabolomics, fecal microbial functional studies and ImP assays	ImP was higher in T2D; mouse/cell studies implicated p38γ–mTORC1 inhibition of insulin signaling	All discovery T2D participants used metformin; validation assessed metabolic phenotype and sex	Tiny discovery cohort; experimental causality cannot be assigned to the human association
Nemet 2020 [33] United States	Discovery and independent prospective validation; 3-year event follow-up	Discovery n = 1,162; independent validation n = 4,000 clinical participants	Untargeted/targeted plasma metabolomics; PAGln; separate microbial and platelet experiments	Higher PAGln associated with CVD and incident MACE; experiments implicated adrenergic receptor–dependent platelet activation	Risk-factor and renal-function adjustment; independent validation	Observational circulating-metabolite associations; experimental mechanisms do not prove that PAGln lowering prevents events
Ruuskanen 2022 [27] Finland, FINRISK	Prospective population cohort; median 15.8-year follow-up	5,572 adults without baseline diabetes; mean age 48.7 years; 432 incident cases	Baseline fecal shotgun metagenomics; registry ascertainment of incident T2D	Several taxa, including Clostridium-related species, predicted incident T2D after adjustment for established risk factors	Multivariable Cox models; geographic validation within Finland	Single baseline stool sample; residual confounding and limited external generalizability
Fromentin 2022 [30] Denmark, France and Germany, MetaCardis	Cross-sectional disease-spectrum comparison	1,241 middle-aged adults: healthy, dysmetabolic without IHD, and IHD at different clinical stages	Gut metagenomics, serum/urine metabolomics and detailed clinical phenotyping	Shared and IHD-related microbial/metabolic features emerged across dysmetabolism, ACS, chronic IHD and heart failure	Explicit consideration of lifestyle, metabolic comorbidities and medication/polypharmacy	Disease-stage ordering is not longitudinal follow-up; residual confounding remains
Talmor-Barkan 2022 [31] Israel with European validation	Case–control multi-omics with external validation	199 patients with ACS in the Israeli discovery cohort; separate non-ACS controls and validation cohorts	Serum metabolomics plus fecal shotgun metagenomics	Person-specific metabolic deviations and microbial features associated with ACS and clinical characteristics/outcomes	Integration of clinical, genetic and environmental features; external cohort validation	Acute illness and treatment can change profiles; observational classifiers do not establish causation
Sayols-Baixeras 2023 [32] Sweden, SCAPIS	Population-based cross-sectional imaging study	8,973 adults without overt ASCVD; age 50–65 years	Fecal shotgun metagenomics, coronary calcium score and CCTA	64 species associated with coronary calcium independently of risk factors; strongest signals included S. anginosus and S. oralis	Adjustment for cardiovascular risk factors; oral-health and inflammatory correlates examined	Imaging association does not establish gut-to-artery causation or predict treatment response

*Abbreviations*: *ACS* acute coronary syndrome, *ASCVD* atherosclerotic cardiovascular disease, *BMI* body mass index, *CCTA* Cardiac Computed Tomography Angiography, *CVD* Coronary vascular disease, *IHD* Ischemic heart disease, *ImP* Imidazole propionate, *PAGln* phenylacetylglutamine, *TMAO* trimethylamine oxide, *MACE* major adverse cardiac event, *T2D* type 2 diabetes

Selection notes: Landmark studies were chosen to represent metagenomic discovery, prospective incident-disease prediction, medication confounding, metabolite biology, multi-omics and directly imaged atherosclerosis. Cross-sectional studies describe prevalent disease, not incident risk. Mechanistic experiments accompanying Koh, Tang and Nemet are distinct from their observational human analyses. Cohorts and subsets can overlap across publications (especially reanalyses and MetaCardis); their sample sizes must not be summed as independent evidence. Associations and statistical adjustment do not establish causal mediation or clinical utility

### Gut Microbiota in Diabetes

Altered microbial composition and function have been reported in IR, metabolic syndrome (MetS), and T2D, but no universal taxonomic signature has emerged. Large shotgun-metagenomic analyses support strain-level and functional differences, including depletion of some butyrate-producing organisms, while also demonstrating substantial variation by population, adiposity, diet, and medication exposure. Changes in the gut virome and phage–bacteria networks have also been described, although their causal significance remains uncertain [4, 5].

### Microbial Metabolites and Metabolic Signaling

**Fig. 1:**
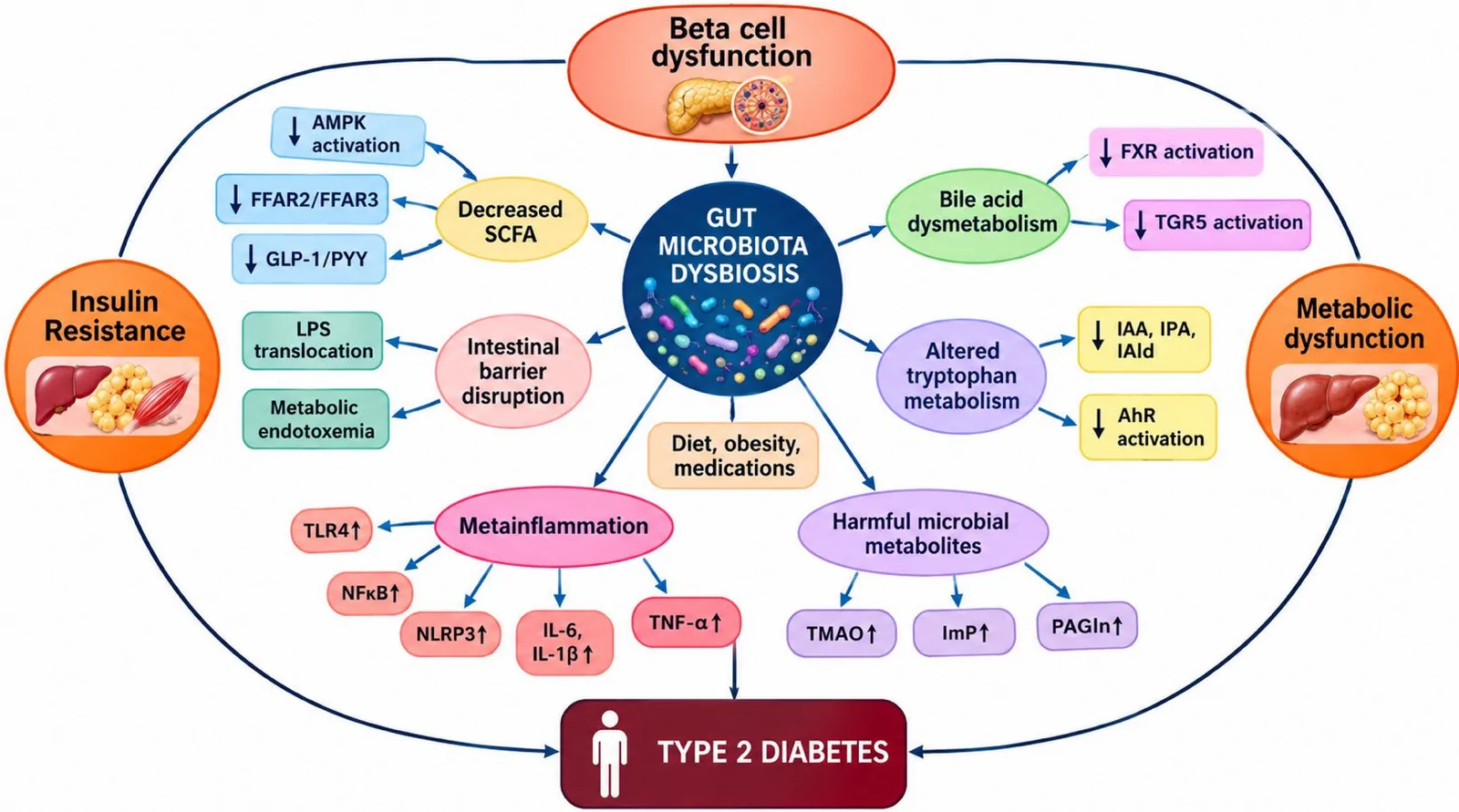
Gut microbiota dysbiosis and metabolic pathways linking the gut microbiota to type 2 diabetes. GM dysbiosis influences glucose metabolism through interconnected pathways involving SCFAs, BA signaling, tryptophan metabolism, other microbial metabolites, intestinal barrier dysfunction, and inflammation. These pathways can promote IR, β-cell dysfunction, impaired GLP-1 signaling, dysglycemia, hepatic gluconeogenesis and systemic inflammation, ultimately contributing to T2D. Dietary factors, obesity, and medications can further modify the gut microbiota and its metabolic functions. Abbreviations: AhR, aryl hydrocarbon receptor; AMPK, AMP-activated protein kinase; BA, bile acid; FFAR2, free fatty acid receptor 2; FFAR3, free fatty acid receptor 3; FXR, farnesoid X receptor; GLP-1, glucagon-like peptide − 1; TGR5, Takeda G protein-coupled receptor 5; IAld, indole − 3-aldehyde; IAA, indole − 3-acetic acid; IL, interleukin; ImP, imidazole propionate; IPA, indole − 3-propionic acid; LPS, lipopolysaccharide; NF-κB, nuclear factor-κB; NLRP3, NOD-like receptor pyrin domain-containing protein 3; PAGln, phenylacetylglutamine; PYY, peptide YY; SCFAs, short-chain fatty acids; T2D, type 2 diabetes; TLR4, Toll-like receptor 4; TMAO, trimethylamine N-oxide; TNF-α, tumor necrosis factor-α

SCFAs (primarily acetate, propionate, and butyrate) are generated by fermentation of dietary fiber. They support mucus production, epithelial energetics, and tight-junction integrity and can limit translocation of microbial products. Through free fatty acid receptors FFAR2/GPR43 and FFAR3/GPR41, enteroendocrine signaling, intestinal gluconeogenesis, and AMP-activated protein kinase, SCFAs may influence glucagon-like peptide − 1 (GLP-1) and peptide YY (PYY) secretion, appetite, glucose handling, lipid metabolism, and inflammation [5] (Fig. 1). These effects are metabolite, tissue, dose, and context-dependent; acetate, for example, cannot be assumed to be uniformly beneficial.

GM-derived tryptophan metabolites, including indole − 3-propionic acid (IPA), indole − 3-acetic acid (IAA), and indole − 3-aldehyde (IAld), signal through the aryl hydrocarbon receptor (AhR) to promote interleukin (IL)− 22 production, regulatory immune responses, and epithelial barrier integrity [35–37]. Higher IPA has been associated with favorable insulin secretion and sensitivity. In contrast, the histidine-derived metabolite imidazole propionate can impair insulin signaling and may blunt metformin action. Phenylacetyl glutamine (PAGln), produced from microbial phenylacetic acid followed by host conjugation, has been associated with T2D and diabetic complications and can signal through adrenergic receptors to influence inflammation and platelet reactivity. These associations do not yet establish clinical utility [4, 38–40]. GM–host cometabolite trimethylamine N-oxide (TMAO) has also been implicated in metabolic dysfunction, with experimental studies suggesting that activation of endoplasmic reticulum stress pathways may contribute to IR [41]. However, the strength and direction of these associations vary among studies, and the causal relevance of individual metabolites in human T2D remains incompletely established.

These findings underscore the importance of microbial metabolic activity, rather than microbial composition alone, in mediating the effects of the GM on host metabolic and immune function.

The GM also transforms primary bile acids (BAs) into secondary BAs through microbial bile salt hydrolase (BSH) activity and 7α-dehydroxylation. Resulting changes in the BA pool alter farnesoid X receptor (FXR) and Takeda G protein coupled receptor 5 (TGR5)/G protein coupled bile acid receptor 1 (GPBAR1) signaling, with downstream effects on GLP-1 secretion, hepatic glucose and lipid metabolism, insulin sensitivity, and energy expenditure. A higher proportion of 12α-hydroxylated BAs has been associated with IR, but BA effects differ by receptor location and metabolic context [42, 43].

### Metainflammation and Intestinal Barrier Dysfunction

**Fig. 2:**
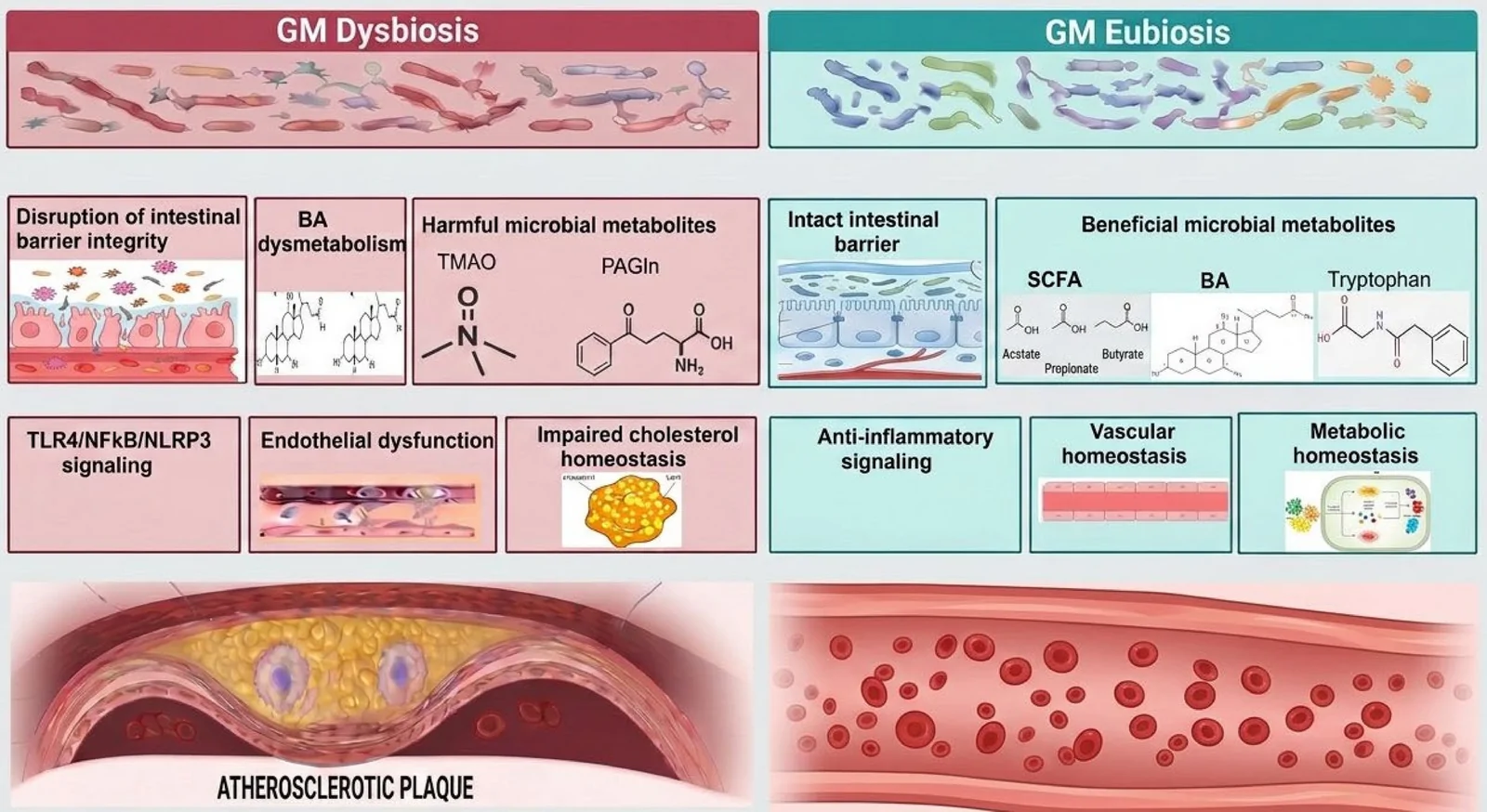
Gut microbiota dysbiosis and metabolic pathways linking the gut microbiota to atherosclerosis. A balanced gut microbiota supports intestinal barrier integrity, anti-inflammatory signaling, and vascular homeostasis through SCFAs, BA, tryptophan, and other microbial metabolites. In contrast, dysbiosis associated with diet, lifestyle, obesity, and other metabolic factors may increase proinflammatory bacteria and microbial products, including LPS, TMA, TMAO, and PAGln. Impaired intestinal barrier integrity promotes translocation of LPS and other microbial products into the circulation, activating TLR4/NF-κB and NLRP3 inflammasome pathways and promoting systemic inflammation. Microbial metabolism of dietary choline, carnitine, and betaine generates TMA, which is converted to TMAO in the liver by FMO3. Altered BA and tryptophan metabolism can disrupt FXR- and TGR5-mediated signaling and immune homeostasis. These interconnected pathways may promote hepatic and vascular inflammation, endothelial dysfunction, macrophage activation, and foam-cell formation, ultimately contributing to atherosclerotic plaque development. The figure depicts proposed mechanisms and does not imply that individual microbial taxa or metabolites are independently causal. Abbreviations: BA, bile acid; GM, gut microbiota; NF-κB, nuclear factor-κB; NLRP3, NOD-like receptor pyrin domain-containing protein 3; PAGln, phenylacetylglutamine; SCFAs, short-chain fatty acid; TLR4, toll like receptor 4; TMAO, trimethylamine -oxide

Barrier dysfunction provides a complementary inflammatory pathway (Figs. 1 and 2). Increased permeability may permit lipopolysaccharide (LPS) and other pathogen-associated molecular patterns (PAMP) to enter the circulation, activating toll like receptor 4 (TLR4)/Myeloid Differentiation Primary Response 88 (MyD88)/nuclear factor-κB (NF-κB) and NOD-like receptor pyrin domain-containing protein 3 (NLRP3) inflammasome signaling in immune and metabolic tissues. Tumor necrosis factor-α (TNF-α), IL-1β, and IL-6 then impair insulin signaling and sustain low-grade metabolic inflammation. However, stool composition is an imperfect proxy for mucosal communities and barrier function and circulating LPS assays have important technical limitations [5, 44, 45].

Alterations in bacteria associated with intestinal barrier maintenance, including *Akkermansia muciniphila* and *Faecalibacterium prausnitzii*, and increased abundance of potentially proinflammatory organisms such as *Escherichia coli*, have been reported in individuals with T2D [46]. Additionally circulating LPS and LPS-binding protein have been reported to be higher in individuals with diabetes than in healthy controls [47].

### Evidence from Human Studies

Human intervention data warrant cautious interpretation. Lean-donor intestinal microbiota transfer has produced short-lived improvements in insulin sensitivity in selected men with MetS, sometimes accompanied by enrichment of butyrate-producing organisms, but findings are inconsistent and may depend on recipient baseline microbiota and donor engraftment [13].

Medication effects complicate interpretation. Metformin alters microbial ecology and BA metabolism; antibiotics, proton-pump inhibitors, laxatives, and statins also leave microbial signatures [48–50]. Studies may therefore misattribute treatment-associated features to T2D. Treatment-naive cohorts, new-user designs, and detailed exposure data are needed. Strain-level resolution is also important because organisms within one species can carry different metabolic genes, while unrelated taxa may provide the same function. Microbial pathways, transcripts, and metabolites may be more reproducible than genus-level abundance, although their output still depends on diet and substrate availability.

### Gut Microbiota and Atherosclerosis

The GM may influence atherogenesis through systemic inflammation, lipid and BA metabolism, endothelial function, platelet reactivity, and microbial metabolites (Fig. 2). Microbial DNA and products have been detected in plaques, but this observation does not establish the presence of viable organisms, a gut origin, or direct plaque colonization [51].

### Intestinal Barrier Dysfunction, LPS, and Inflammation

Barrier disruption and circulating LPS can activate TLR4/MyD88/NF-κB signaling in macrophages, endothelial cells, hepatocytes, and vascular smooth muscle cells. The resulting cytokines, chemokines, adhesion molecules, inflammasome activation, and smooth-muscle phenotypic changes may promote monocyte recruitment, foam-cell formation, endothelial dysfunction, and plaque progression [51, 52] (Fig. 2).

### BA Metabolism and Signaling

The GM plays a central role in BA metabolism, which influences both lipid homeostasis and vascular inflammation. Primary BAs, cholic acid and chenodeoxycholic acid (CDCA), are synthesized from cholesterol in the liver and undergo extensive enterohepatic recycling. Approximately 95% of intestinal BAs are reabsorbed, while the fraction that escapes absorption is modified by the GM through BSH, 7α-dehydroxylation, oxidation, and epimerization to generate secondary BAs, including deoxycholic acid and lithocholic acid [53].

Alterations in GM composition and BSH activity may modify the BA pool and alter FXR signaling, with potential consequences for cholesterol metabolism and atherosclerosis [43, 53]. Because microbial conversion of primary to secondary BAs contributes to the generation of TGR5 ligands, dysbiosis-associated changes in BA metabolism may influence both metabolic and vascular pathways. However, the effects of individual BAs and FXR/TGR5 signaling are tissue- and context-dependent and require further clarification in human atherosclerosis [53, 54].

### TMAO and ASCVD Risk

TMAO is generated when microbial enzymes convert dietary choline, phosphatidylcholine, and L-carnitine to trimethylamine (TMA), which hepatic flavin-containing monooxygenase 3 (FMO3) converts to TMAO. Experimental studies implicate TMAO in altered cholesterol transport, endothelial dysfunction, vascular inflammation, foam-cell formation, and heightened platelet responsiveness. Prospective studies and meta-analyses associate higher circulating TMAO with cardiovascular events and mortality [5, 51, 55]. Interpretation requires caution: diet, hepatic FMO3 activity, microbial enzyme capacity, and, in particular, kidney function are strong determinants of circulating concentrations. Reverse causation and residual confounding remain plausible, and lowering TMAO has not yet been shown to reduce ASCVD events.

### SCFAs and Atheroprotection

SCFAs, particularly acetate, propionate, and butyrate, may exert anti-inflammatory and potentially atheroprotective effects through inhibition of histone deacetylases and activation of G protein-coupled receptors, including FFAR2 (GPR43) and FFAR3 (GPR41) [51]. Preclinical studies suggest that SCFAs can suppress NF-κB signaling and NLRP3 inflammasome activation, reduce endothelial expression of adhesion molecules, and decrease monocyte recruitment and foam-cell formation [56, 57] (Fig. 2).

However, evidence for atheroprotection in humans remains limited. In a randomized, double-blind, placebo-controlled trial, oral propionate supplementation for 8 weeks reduced low density lipoprotein cholesterol (LDL-C), total, and non-high density lipoprotein cholesterol (NonHDL-C) in individuals with elevated baseline LDL-C [19]. In contrast, evidence supporting direct anti-atherosclerotic effects of butyrate remains largely limited to experimental models, and its clinical relevance requires further investigation [56, 58–61].

### Other GM Metabolites and Cardiovascular Risk

Other metabolites may be equally informative (Fig. 2). PAGln can enhance platelet responsiveness through adrenergic receptors and has been associated with thrombosis and adverse cardiovascular outcomes [33, 41, 62]. Microbial indole derivatives, including IPA, IAA, and IAld, can activate the AhR and influence intestinal barrier integrity, immune-cell differentiation, oxidative stress, and inflammation. Several indole metabolites exhibit anti-inflammatory and antioxidant properties and may therefore have vasculoprotective effects. In contrast, the host kynurenine pathway is activated by inflammatory signaling and generates metabolites that have been associated with endothelial dysfunction and cardiovascular risk. Importantly, AhR effects vary according to the specific ligand, tissue, and physiologic context [63]. Indoxyl sulfate and p-cresyl sulfate, produced through microbial–host metabolism and retained in chronic kidney disease, may promote oxidative stress and endothelial dysfunction [64]. These pathways argue against labeling the microbiome globally as beneficial or harmful; functional output is the more relevant unit of analysis.

Animal transfer and gnotobiotic experiments support biological plausibility, but human evidence is predominantly observational (Table 2). Statins, metformin, diet, smoking, bowel transit, obesity, and renal function can alter both microbiota and ASCVD risk. Robust translation therefore requires repeated metabolite measurement, medication-aware analyses, direct assessment of microbial genes and pathways, and clinical rather than surrogate vascular outcomes.

Microbial effects may also differ by disease stage. Endotoxemia could influence endothelial activation and monocyte recruitment; altered cholesterol and BA handling could affect plaque growth; and TMAO- or PAGln-related platelet hyperreactivity could influence thrombosis after plaque disruption. Human studies rarely distinguish these stages. Moreover, oral and periodontal microbes may contribute to circulating products and correlate with vascular disease. Bacterial DNA in plaques therefore does not establish a gut-to-plaque route without source tracking and contamination controls.

### Gut Microbiota- Diabetes and Atherosclerosis Connection

**Fig. 3:**
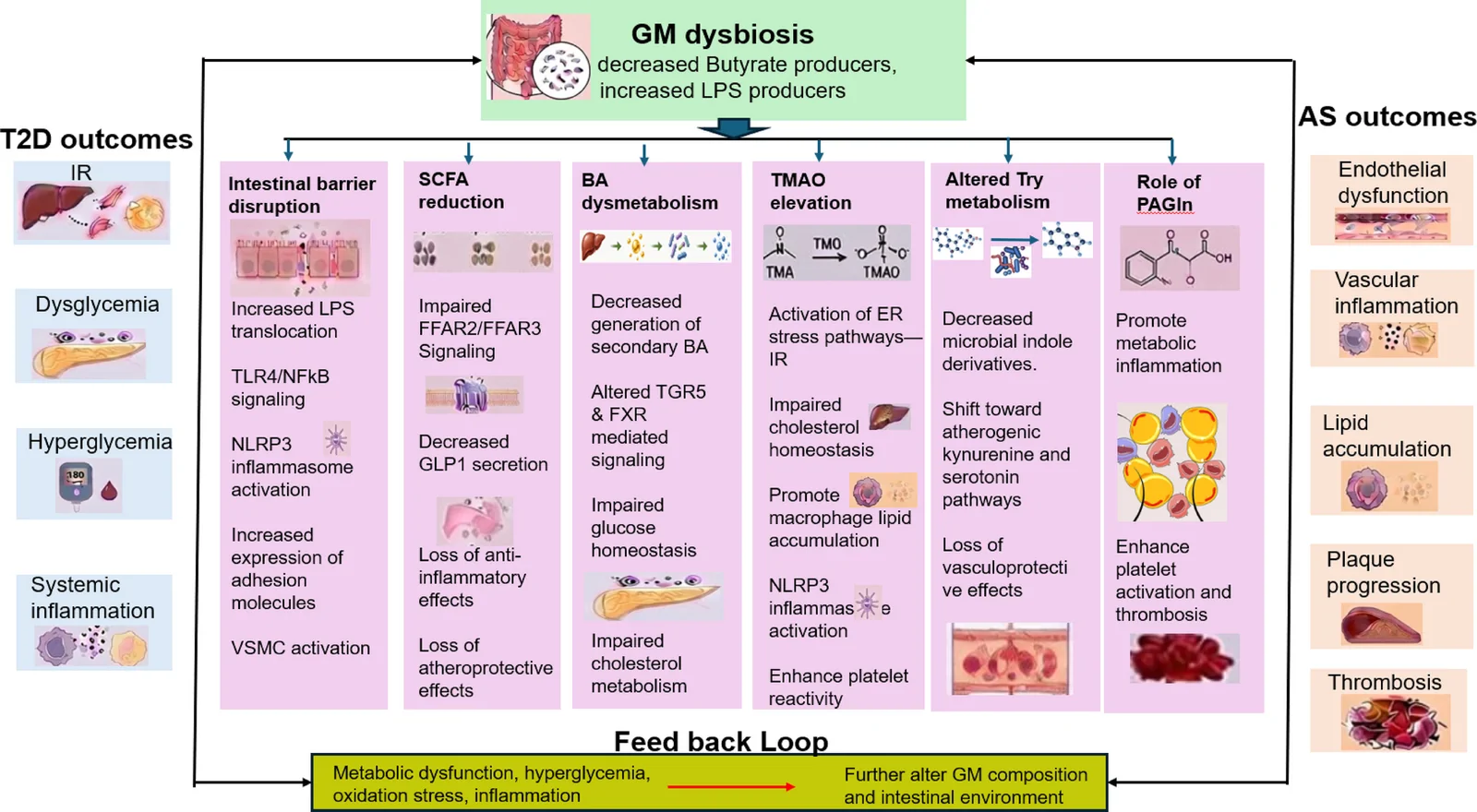
The gut microbiota–type 2 diabetes–atherosclerosis axis. GM dysbiosis contributes to T2D and atherosclerosis through alterations in microbial metabolites, intestinal barrier integrity, and inflammatory signaling. Reduced SCFA production, increased LPS translocation, altered BA metabolism, and increased TMAO production promote insulin resistance, systemic inflammation, endothelial dysfunction, and vascular inflammation. Dysbiosis may also alter GLP-1 secretion, further affecting glucose homeostasis. In turn, hyperglycemia, insulin resistance, and chronic inflammation associated with T2D can exacerbate GM dysbiosis, creating a feed-forward cycle that promotes atherosclerotic plaque development and progression. Collectively, these bidirectional interactions link GM dysbiosis, metabolic dysfunction, and cardiovascular disease. Abbreviations: AS-atherosclerosis; BA, bile acids; ER-endoplasmic reticulum FXR- Farnesoid X receptor; FFAR2, free fatty acid receptor 2; FFAR3, free fatty acid receptor 3; GLP-1, glucagon-like peptide − 1; GM, gut microbiota; IR- insulin resistance; LPS, lipopolysaccharide; NF-κB, nuclear factor-κB; NLRP3, NOD-like receptor pyrin domain-containing protein 3;PAGln, phenylacetylglutamine; SCFAs, short-chain fatty acids; TGR5, Takeda G protein-coupled receptor 5; T2D, type 2 diabetes; TLR4, Toll-like receptor 4; TMAO, trimethylamine oxide; Try, tryptophan; VSMC- vascular smooth muscle cell

T2D and atherosclerosis share a microbiota-associated network rather than two independent pathways. IR and hyperglycemia can impair barrier function and reshape microbial ecology; microbial products and metabolites may then amplify systemic and vascular inflammation (Fig. 3). LPS/TLR4/NF-κB and inflammasome signaling links adipose-tissue inflammation and impaired insulin action with endothelial activation and plaque progression. Loss of butyrate-producing capacity may weaken the barrier and anti-inflammatory signaling, whereas altered BA signaling affects both glucose regulation and cholesterol metabolism [65–71].

Microbial metabolites provide an additional point of convergence. TMAO has been associated with IR and, independently, with impaired cholesterol homeostasis, foam-cell formation, endothelial dysfunction, and platelet hyperreactivity. PAGln provides additional links between metabolism and thrombosis. Similarly, BA signaling through FXR and TGR5 can influence both glucose metabolism and vascular inflammation. Conversely, reduced SCFA production may diminish GLP-1 secretion, insulin-sensitizing effects, and anti-inflammatory signaling, potentially promoting both metabolic and vascular dysfunction [5, 71].

T2D, dietary patterns, and kidney disease may increase exposure to the harmful metabolites, which can influence platelet and vascular biology. This cardio–renal–metabolic context is important because reduced renal clearance raises TMAO, PAGln, indoxyl sulfate, and p-cresyl sulfate while chronic kidney disease independently increases ASCVD risk. Renal function is therefore both a mechanistic component and a major confounder [64, 72].

The shared-axis model also explains why causality is difficult to assign. Diet, adiposity, glycemic control, drugs, and vascular disease simultaneously affect the microbiome and clinical outcomes. Cross-sectional taxonomic associations cannot determine directionality, and similar functional pathways may be supplied by different organisms in different individuals. Longitudinal, multi-omic studies should test whether microbial genes and metabolites mediate the excess vascular risk of T2D beyond established risk factors.

Biomarkers, mediators, and therapeutic targets should be distinguished. A microbial feature may predict disease without causing it; a causal mediator may be difficult or unsafe to modify; and changing a microbial marker may not improve outcomes. A clinically relevant target requires temporal precedence, mechanistic specificity, reproducible human evidence, successful perturbation, and outcome improvement without unacceptable off-target effects.

### Therapeutic Implications

**Fig. 4:**
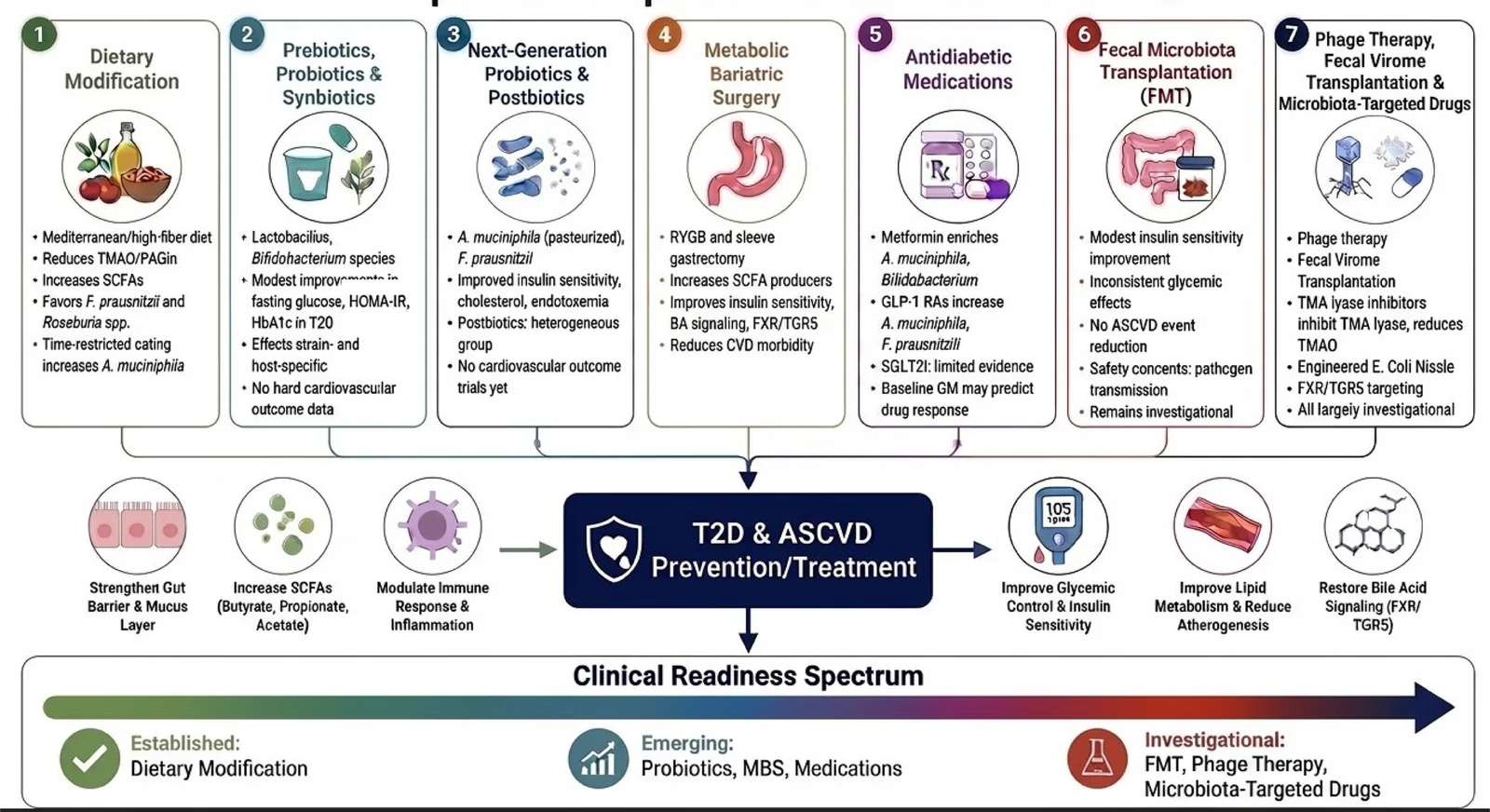
Therapeutic manipulation of gut microbiota. Targeted modulation of the GM represents a potential strategy for improving metabolic health and reducing cardiovascular risk. Dietary modification, prebiotics, probiotics, synbiotics, FMT, FVT, and microbiota-targeted pharmacologic approaches may restore microbial diversity and function, enhance beneficial metabolites such as SCFAs, improve intestinal barrier integrity, and reduce inflammation. Modulation of BA signaling, TMAO production, and GLP-1 pathways may further improve glucose and lipid metabolism and attenuate atherosclerotic processes. These approaches may therefore offer therapeutic opportunities for interrupting the GM–T2D–atherosclerosis axis. Abbreviations: ASCVD, atherosclerotic cardiovascular disease; BA, bile acid; CVD, cardiovascular disease; FXR, Farnesoid X receptor; FMT- fecal microbiota transplantation; FVT, fecal virome transplantation; GLP-1, glucagon-like peptide − 1; GLP-1RA, glucagon-like peptide − 1 receptor agonist; GM, gut microbiota; HbA1c, Hemoglobin A1c; HOMA-IR, Homeostatic Model Assessment for Insulin Resistance; MBS, metabolic bariatric surgery; PAGln, phenylacetylglutamine; RYGB, Roux-en-Y gastric bypass; SCFAs, short-chain fatty acids; SGLT2I, Sodium-Glucose Cotransporter-2 inhibitors; TGR5, Takeda G protein-coupled receptor 5; TMA, Trimethylamine; TMAO, trimethylamine N-oxide; T2D, type 2 diabetes

Microbiota-directed treatment can target the ecological community, its substrates, or specific microbial products (Fig. 4, Table 1). At present, no microbiome test or intervention is recommended specifically to prevent T2D or ASCVD events; standard risk-factor management remains essential.

### Dietary Modification

Diet has the strongest combination of safety, feasibility, and clinical relevance. Plant-rich Mediterranean-style patterns, whole grains, legumes, nuts, fruits, vegetables, and fermentable fiber generally favor SCFA-producing functions and are independently associated with cardiometabolic benefit. In contrast, low-fiber dietary patterns rich in ultra-processed foods and selected animal-derived TMA precursors may favor adverse metabolic outputs. Responses are individualized, and benefits cannot be attributed solely to the microbiome. Precision-nutrition studies integrating baseline microbiota with postprandial and metabolic phenotypes are promising but not yet ready for routine use [4, 34, 44, 68, 73–76].

## Prebiotics, Probiotics, Synbiotics

Trials of conventional probiotics, prebiotics, and synbiotics report small, heterogeneous improvements in fasting glucose, IR, HbA1c, lipids, blood pressure, or inflammatory markers [20, 68, 77–81].

Effects depend on strain, substrate, dose, duration, background diet, and host phenotype; class-wide claims are inappropriate. No trial has demonstrated a reduction in microvascular or cardiovascular events [78, 82–85].

### Next Generation Probiotics (NGPs) and Postbiotics

*Akkermansia muciniphila* and *Faecalibacterium prausnitzii* are among the most extensively studied NGP candidates and have been associated with metabolic health [86, 87]. Evidence linking NGPs with atherosclerosis remains predominantly preclinical. Higher abundance of *F. prausnitzii* has been associated with lower coronary artery disease risk, while experimental studies suggest that supplementation may reduce intestinal LPS production and strengthen mucosal barrier function [88].

Postbiotics, comprising non-viable microorganisms, microbial components, or metabolites, offer a potentially safer alternative to live microbial administration. Pasteurized *A. muciniphila* has shown proof-of-concept improvements in insulin sensitivity and selected metabolic measures, but larger confirmatory and outcome trials are required [17, 80, 89]. However, postbiotics represent a heterogeneous group, and biological effects vary according to the microbial species and component administered [90]. Concerns include strain-specific efficacy, manufacturing standardization, long-term safety, and, for live organisms, potential opportunistic infection and antimicrobial-resistance gene transfer. No trials have yet demonstrated hard cardiovascular or microvascular outcome benefits [80, 86, 89, 91].

### Metabolic Bariatric Surgery

Metabolic bariatric surgery produces large changes in microbial ecology, BA signaling, enteroendocrine hormones, and metabolism. Some microbial changes correlate with diabetes remission and improvement in several atherosclerotic risk factors and transfer experiments suggest partial mediation; nevertheless, weight loss, caloric restriction, altered anatomy, and medication changes prevent attribution of clinical benefit to the GM alone [23, 92–96].

### Antidiabetic Medication

Glucose-lowering drugs also modify the GM [70, 97]. Metformin has the strongest evidence, including enrichment of selected SCFA-associated taxa, altered BA metabolism, and transfer of improved glucose tolerance to germ-free mice [48, 49]. GLP-1 receptor agonists and sodium–glucose cotransporter-2 inhibitors produce variable changes in the GM; however, human evidence that these changes mediate cardiovascular benefits remains limited. Baseline microbiota may predict short-term drug response, although proposed signatures have not been prospectively validated. Medication exposure must therefore be treated as both a potential mechanism and a confounder [48, 98–103].

### Fecal Microbiota Transplantation

Fecal microbiota transplantation (FMT) for metabolic disease remains experimental. Trials show variable, often transient donor engraftment and at most modest effects on insulin sensitivity, with inconsistent changes in weight, HbA1c, and lipids and no demonstrated reduction in ASCVD events [14, 16, 41, 49, 104–110]. Donor effects, recipient ecology, preparation, route, dosing, and durability are unresolved. Pathogen transmission and antimicrobial-resistance genes require rigorous donor screening and long-term surveillance. [48, 105, 110, 111].

### Gut virome Transplantation and Phage therapy

The gut virome, chiefly bacteriophages, shapes bacterial community structure and is altered in metabolic disease, prompting interest in fecal virome transplantation (FVT) and defined phage therapy as more targeted alternatives to broad microbiota manipulation [112–115]. In animal models, FVT from metabolically healthy donors reshapes the bacteriome and metabolome and improves glucose regulation, and phage cocktails improve metabolic phenotypes alongside expansion of SCFA producers and reduced endotoxin-driven inflammation [113, 116–118]. Human data are sparse: a randomized pilot in MetS found sterile fecal filtrate transfer safe and transiently phageome-altering but without glycemic benefit [18]. No trials have examined atherosclerotic outcomes, and vascular applications such as depleting TMA- or endotoxin-producing taxa remain conceptual, with narrow host range, resistance, immune clearance, manufacturing complexity, and unknown long-term safety as further barriers [115, 119, 120].

### Microbiota-Targeted Drugs

Microbiota-targeted drugs modify microbial metabolic pathways rather than community composition. The choline analogue 3,3-dimethyl-1-butanol (DMB) and related non-lethal TMA-lyase inhibitors reduce TMAO production and atherosclerotic or thrombotic phenotypes in experimental models [121]. Engineered *Escherichia coli* Nissle 1917 strain, producing propionate and butyrate shows metabolic and cardiovascular effects in animal models [121]. Similarly, manipulation of BA signaling through host FXR and TGR5 pathways represents another strategy targeting the microbiota–metabolite–host signaling axis [122]. However, these approaches remain predominantly preclinical, with limited human efficacy and safety data [121, 122].

Overall, dietary modification currently offers the best balance of feasibility, safety, and scalability despite individualized responses; probiotics, prebiotics, synbiotics, next-generation probiotics, and postbiotics show modest metabolic effects without cardiovascular outcome data; metabolic-bariatric surgery and antidiabetic drugs reliably alter the microbiota, though the causal contribution of those shifts is unclear; and FMT, FVT, phage therapy, and microbiota-targeted drugs remain investigational despite greater mechanistic precision.

### Limitations

This review has several limitations. First, it was designed as a narrative review and therefore did not follow a formal systematic review or meta-analysis methodology. Although PubMed (from database inception) and Google Scholar were searched through August 2026 using the keyword combinations described in the Methods related to gut microbiota, T2D, and atherosclerosis, study selection was based on relevance and methodological rigor rather than a prespecified systematic screening and risk-of-bias assessment. Consequently, the possibility of selection bias and omission of relevant studies cannot be excluded.

Second, interpretation of the available evidence is limited by substantial heterogeneity across studies. Most human data are observational and cross-sectional, and no consistent disease-specific microbial signature has emerged. The relatively reproducible findings, including reduced diversity and depletion of butyrate producers, are nonspecific to T2D or ASCVD [48]. Confounding by age, BMI, diet, bowel habits, hepatic FMO3 genotype, and drugs such as metformin and statins can attenuate or abolish reported case–control differences, and interindividual compositional variation exceeds the variation attributable to individual dietary factors [121, 123–125]. Methodological heterogeneity in sample collection, extraction, sequencing, and bioinformatics further undermines reproducibility, and robust associations may require far larger cohorts than those available in most existing studies.

Third, the predominantly associative nature of the human literature limits causal inference. Experimental studies using fecal microbiota transplantation and gnotobiotic models suggest that microbial communities can influence metabolic and vascular phenotypes, but findings from animal models may not fully translate to humans. Conversely, obesity, hyperglycemia, dyslipidemia, diet, and medications can themselves alter the gut microbiota, making it difficult to distinguish cause from consequence [48].

Finally, evidence for microbiota-directed therapies remains limited. Therapeutically, FMT yields variable and often transient effects, probiotics and postbiotics are strain- and formulation-specific with mostly surrogate endpoints, and enzyme inhibitors and engineered organisms remain preclinical, with no trials showing reduced cardiovascular events. Marked interindividual variability, including FMO3-driven differences in TMAO production, argues against universal microbiota-directed interventions, and long-term efficacy, durability, safety, and unintended consequences remain poorly defined [105, 121]. These limitations underscore the need for large, longitudinal, multi-omics studies and adequately powered randomized controlled trials with clinically meaningful metabolic and cardiovascular outcomes before microbiota-directed approaches can be incorporated into routine clinical practice.

### Knowledge Gaps

Several fundamental questions remain unresolved. It is not yet clear which microbial alterations or microbial functions are directly involved in the development and progression of T2D and atherosclerosis, as opposed to representing consequences of metabolic disease or its treatment. In particular, the relative contributions of microbial composition, microbial metabolites, and host–microbe interactions to insulin resistance, dyslipidemia, inflammation, and atherosclerotic disease remain incompletely defined.

The determinants of interindividual differences in microbiota-related metabolic and cardiovascular risk also require further investigation. Host genetics, diet, metabolic phenotype, and medication exposure may modify microbial functions and their effects on the host. For example, variation in hepatic FMO3 activity may influence TMAO production and cardiovascular risk [105, 121]. It remains unclear whether these factors can be used to identify biologically and clinically meaningful subgroups that would respond differently to microbiota-directed interventions.

Although reduced microbial diversity and depletion of butyrate-producing bacteria have been associated with metabolic and cardiovascular disease, these findings are not disease-specific and their value for individual risk stratification or treatment selection remains uncertain [48, 105].

Finally, it remains unknown whether modifying the gut microbiota can produce sustained improvements in clinically meaningful outcomes beyond established approaches to T2D and ASCVD. The optimal microbial targets, therapeutic strategies, duration of treatment, and patient populations remain undefined. In particular, whether microbiota-directed interventions can reduce atherosclerotic progression or cardiovascular events, rather than only altering intermediate biomarkers, remains an important unanswered question.

### Future Directions

Future work should move the field from association to causality and from surrogate markers to clinically meaningful outcomes. Priorities include large, longitudinal, multi-ethnic cohorts using standardized shotgun metagenomics, metabolomics, and deep host phenotyping, with rigorous control for diet, medications, and other confounders, strengthened by Mendelian randomization, mediation analysis, and gnotobiotic models. Mechanistic work should shift from taxonomy toward function, targeting specific enzymes and pathways such as TMA lyases, bile salt hydrolases, and SCFA- and tryptophan-related pathways [48, 121, 125].

Therapeutically, adequately powered outcome-driven randomized trials are needed for precision interventions, strain-defined next-generation probiotics and postbiotics, engineered microorganisms, microbial enzyme or metabolite inhibitors, and optimized FMT, incorporating baseline microbiota and pharmaco-microbiomic profiling to identify likely responders and enable microbiome-guided therapy and personalized nutrition. Clinical implementation should await reproducible evidence of efficacy and safety across diverse populations. Progress will also depend on methodological standardization, validated mechanistic biomarkers, and systematic evaluation of long-term safety, durability, and regulatory requirements for live-microbial and metabolite-targeted therapies [48, 121, 125].

## Conclusions

Taken together, the evidence reviewed here supports GM dysbiosis as a biologically plausible shared contributor to T2D and atherosclerosis, acting through overlapping pathways that include loss of intestinal barrier integrity with metabolic endotoxemia, inflammation driven by TLR4/NF-κB and inflammasome signaling, and altered production of microbial metabolites such as SCFAs, BAs, TMAO, PAGln, and tryptophan-derived indoles. Nonetheless, most human data remain observational and confounded, causal evidence is incomplete, and responses to microbiota-directed interventions are heterogeneous; consequently, the extent to which these microbial alterations are causal or modifiable determinants of T2D and atherosclerotic disease remains uncertain.

Evidence for microbiota-directed interventions is also emerging but remains insufficient to support routine clinical use. Studies of dietary modification, probiotics, postbiotics, FMT, and metabolite- or enzyme-targeted approaches have shown variable effects, and evidence for durable improvements in clinically meaningful metabolic or cardiovascular outcomes is limited. Importantly, established strategies—including Mediterranean-style nutrition, physical activity, smoking avoidance, weight management, glycemic control, blood-pressure treatment, and evidence-based lipid lowering—improve cardiovascular and metabolic outcomes irrespective of microbiota mediation. Accordingly, microbiota-directed therapies should currently be viewed as adjuncts rather than replacements for guideline-directed risk-factor management. Safety considerations also differ by modality: live microbial products may raise concerns regarding infection and antimicrobial resistance; phage-based approaches may select for phage resistance; enzyme inhibitors may have unintended effects on host physiology; and engineered organisms require appropriate containment and mechanisms for reversibility. Future studies should prioritize rigorous causal evaluation, standardized intervention strategies, and clinically meaningful cardiovascular and metabolic outcomes to determine whether targeting the GM can provide incremental benefit beyond established therapies.

## Key References

- Byndloss M, Devkota S, Duca F, Niess JH, Nieuwdorp M, Orho-Melander M, et al. The Gut Microbiota and Diabetes: Research, Translation, and Clinical Applications—2023 Diabetes, Diabetes Care, and Diabetologia Expert Forum. Diabetes Care. 2024;47(9):1491–508.
  - ○ This expert consensus review summarizes the current state of the science on the role of the GM in diabetes, including mechanistic insights, translational research, and clinical applications.
- Sasidharan Pillai S, Ashraf AP. Gut Microbiota and Metabolic Health: From Dysbiosis to Therapeutics. Diabetes Ther. 2026;17(4):483–97.
  - ○ This review synthesizes current evidence on GM dysbiosis and metabolic health, emphasizing functional mechanisms beyond taxonomic associations and potential microbiota-targeted therapeutic strategies.
- Salazar J, Morillo V, Suárez MK, Castro A, Ramírez P, Rojas M, et al. Role of Gut Microbiome in Atherosclerosis: Molecular and Therapeutic Aspects. Curr Cardiol Rev. 2023;19(4): e020223213408.
  - ○ This review summarizes the molecular mechanisms linking the GM with atherosclerosis and discusses emerging microbiota-targeted therapeutic strategies.
- Sasidharan Pillai S, Gagnon CA, Foster C, Ashraf AP. Exploring the Gut Microbiota: Key Insights Into Its Role in Obesity, Metabolic Syndrome, and Type 2 Diabetes. J Clin Endocrinol Metab. 2024;109(11):2709–19.
  - ○ This mini review examines the roles and mechanisms of the GM in obesity, MetS, and T2D and discusses potential therapeutic strategies targeting the microbiota.
- Snelson M, Muralitharan RR, Liu CF, Markó L, Forslund SK, Marques FZ, et al. Gut-Heart Axis: The Role of Gut Microbiota and Metabolites in Heart Failure. Circ Res. 2025;136(11):1382–406.
  - ○ This review examines the interplay between the GM, microbial metabolites, and heart failure, with emphasis on dietary influences and emerging gut-targeted therapeutic approaches.
- Tripathy M, Swain J, Das S. The Association of Diabetes and Atherosclerosis: Understanding the Molecular Mechanism of the Double Burden. Curr Med Chem Published online June 17, 2026. 2026 Jun 19;33.
  - ○ This review analyzes the established and emerging mechanisms linking type 2 diabetes and atherosclerosis, providing an integrated understanding of their shared molecular pathways.
- Zhu G, Man J. Gut microbiota-derived metabolites in cardiovascular disease: mechanisms, disease-specific roles, and translational opportunities. Front Cardiovasc Med. 2026; 13:1,884,283.
  - ○ This review examines how gut microbiota-derived metabolites, particularly TMAO, SCFAs, PAGln, bile acids, and tryptophan-derived metabolites—affect cardiovascular disease through pathways involving inflammation, endothelial dysfunction, lipid metabolism, thrombosis, and vascular/cardiac remodeling. It also evaluates the translational potential and limitations of targeting these microbial metabolites, emphasizing that most evidence remains mechanistic or observational and that stronger causal and interventional evidence is needed before clinical implementation.
- Sammad A, Khan S, Zhang YY, Bai XL, Zhu X, Yin K. Gut Microbial Dysbiosis in Type 2 Diabetes Patients with Concurrent Atherosclerosis. Curr Atheroscler Rep. 2026;28(1):52.
  - ○ The review examines gut microbiome dysbiosis specifically in patients with type 2 diabetes and concurrent atherosclerosis, highlighting population-specific microbial signatures associated with disease severity.
- Chen Y, Jin D, Han X, Liu X, Liu Y, Wang L. Gut microbiota in type 2 diabetes mellitus: mechanistic links between dysbiosis, insulin resistance, and chronic low-grade inflammation. Front Endocrinol (Lausanne). 2026; 17:1,856,667.
  - ○ This narrative review examines how gut microbiota dysbiosis may contribute to type 2 diabetes through impaired intestinal barrier function, microbial metabolites, chronic low-grade inflammation, and disrupted insulin signaling.
- Wen D, Wang X, He Y, Hao X, Huang W, Zhou H, et al. Pharmacomicrobiomics: Exploring the role of gut microbiota in type 2 diabetes and antidiabetic drug mechanisms. Nutr Metab Cardiovasc Dis. 2026;36(2):104,379.
  - ○ This review examines the bidirectional interactions between the gut microbiota and antidiabetic medications, including metformin, SGLT2 inhibitors, GLP-1 receptor agonists, and α-glucosidase inhibitors.
- Chica Cardenas LA, Leonard MM, Baldridge MT, Handley SA. Gut virome dynamics: from commensal to critical player in health and disease. Nat Rev Gastroenterol Hepatol. 2026;23(2):126–44.
  - ○ This review examines the gut virome—particularly bacteriophages and eukaryotic viruses—as an important regulator of the gut microbiota, host immunity, and metabolic health.
- Fusco W, Adolph T, Cammarota G, Gasbarrini A, Ianiro G, Tilg H. Gut microbiota and atherosclerosis. Gut. 2026;75(5):1067–77.
  - ○ This review examines how the gut microbiota contributes to atherosclerosis through host–microbe interactions, microbial metabolites, inflammation, lipid metabolism, and vascular aging.
- Ortega-Reyes D, Asami S, Suda W, Ohno H. Host Genetics, Diet, and the Gut Microbiome: Addressing Methodological and Reproducibility Challenges in Human Studies. J Nutr. 2026;156(8):101,646.
  - ○ This narrative review focuses on the complex interplay among host genetics, diet, and the gut microbiome, with particular emphasis on why findings from human studies are often difficult to reproduce.

## Data Availability

No datasets were generated or analysed during the current study.

## Declarations

### Prior Publication/Presentation

Part of the contents of this manuscript was published in JCEM (https://doi.org/10.1210/clinem/dgae499) and Diabetes Therapy (https://doi.org/10.1007/s13300-026-01851-x) and presented at the Annual Conference of the Endocrine Society on July 14, 2025, in San Francisco, CA, USA.
